# Effects of different amino acid levels and a carvacrol–thymol blend on growth performance and intestinal health of weaned pigs

**DOI:** 10.1186/s40104-022-00674-7

**Published:** 2022-03-08

**Authors:** Yanan Wang, Zhipeng Yang, Yuanfei Zhou, Jiajian Tan, Haiqing Sun, Defa Sun, Yuyun Mu, Jian Peng, Hongkui Wei

**Affiliations:** 1 Department of Animal Nutrition and Feed Science, College of Animal Science and Technology, Huazhong Agricultural University, Wuhan, 430070 China; 2 Guangxi Yangxiang Co., Ltd, Guigang, 537000 China; 3 Novus International Trading (Shanghai) Co. Ltd, Shanghai, 200080 China; 4 The Cooperative Innovation Center for Sustainable Pig Production, Wuhan, 430070 China

**Keywords:** Amino acids, Antioxidant capacity, Carvacrol and thymol, Intestinal health, Plant extracts, Weaned pigs

## Abstract

**Background:**

Over the past years, antibiotic growth promoter had been restricted in animal husbandry production in many countries because of antimicrobial resistance and foodborne antibiotic residues. However, the problems of poor intestinal health and low growth efficiency of piglets have not been solved completely in an antibiotic-free diet, and it is urgent to explore alternatives to antimicrobial growth promoters.

**Methods:**

Here, a total of 532 weaned pigs were assigned to one of 4 treatments, the low amino acid (AA) level diet (d 1 to d 14 is 1.35%, d 15 to d 42 is 1.25%) (Low AA), the low AA level diet supplementation with a carvacrol–thymol blend (50 mg carvacrol and 50 mg thymol/kg of diet) (CB) (Low AA+CB), the high AA level diet (d 1 to d 14 is 1.50%, d 15 to d 42 is 1.40%) (High AA), and the high AA level diet supplementation with a CB (High AA+CB), respectively. Then we measured growth performance and intestinal health indicators of weaned pigs.

**Results:**

Results showed that high AA level significantly reduced plasma urea nitrogen, plasma Interleukin-6 (IL-6) and fecal lipocalin-2 contents (*P* <  0.05), significantly increased the relative abundance of fecal *Lactobacillus* and *Enterococcus*, and had a trend to increase the fecal secretory immunoglobulin A (sIgA) and mucin 2 (MUC 2) contents (*P* <  0.05) in piglets, thereby alleviating the diarrhea of piglets and reducing the feed conversion ratio (FCR) of piglets during d 1~14 after weaning. Dietary supplementation with CB significantly increased the activity of plasma antioxidant enzymes T-SOD and GSH-px (*P* <  0.05), while significantly reduced plasma malondialdehyde (MDA), plasma interleukin-1β (IL-1β), plasma endotoxin and *D*-lactic acid contents (*P* <  0.05). Meanwhile, CB significantly decreased fecal lipocalin-2 contents and the abundance of fecal *Escherichia coli* (*P* < 0.05). Thus, we hypothesis that dietary supplementation with CB significantly increased the average daily gain (ADG) of piglets (*P* < 0.05) during d 1~14 after weaning through promoting intestinal health.

**Conclusion:**

These results suggest that high AA level and dietary supplementation with CB improved the growth performance of weaned pigs in an antibiotic-free diet by improving AA metabolism and intestinal antioxidant capacity.

## Introduction

Antibiotics are usually used to prevent and treat animal diseases which play an important role in animal husbandry production [1]. There are approximately 75% of antibiotics used in animal husbandry production worldwide since antibiotics were discovered to accelerate pig and chicken growth [2]. In recent years, many countries have realized that the abuse of antibiotic had led to antimicrobial resistance and foodborne antibiotic residues, and began to implement antibiotic free feeding [3]. Following the European Union and the United States, China began to ban antimicrobial growth promoters from July 1, 2020. However, the problems of poor intestinal health and low growth efficiency of weaned pigs have not been solved completely in an antibiotic-free diet [4]. Therefore, it is urgent to explore alternatives to antimicrobial growth promoters.

Dietary AA are metabolized in the small intestine, and about one third of the dietary essential AA are consumed through the first pass metabolism of the intestine [5, 6]. Porcine small intestine bacteria can quickly utilize lysine, arginine, threonine, and glutamic acid [7, 8]. Slightly less than lethal doses of antibiotic could significantly reduce total bacterial abundance [9]. Thus, the increase in the abundance of microbes induced by antibiotic-free diet in the small intestine may lead to an increase in AA requirements. Moreover, the requirement of lysine or methionine as energy sources or to support the immune system may also be significantly higher than that in an antibiotic-free diet [10]. Hence, high AA level may be an effective measure to improve the growth performance of weaned pigs fed with antibiotic-free diet [10–12].

Carvacrol and thymol are both phenolic monoterpenoids, which are extracted form origanum vulgare. Carvacrol and thymol have been proven to exert a variety of physiological activities, such as anti-microbial, anti-inflammatory, anti-oxidative, immune modulation, and improving intestinal morphology and intestinal mucosal integrity [13–16]. Thus, carvacrol and thymol are widely used as a substitute for antibiotics in animal diets [16–18]. Our previous studies also found that dietary supplementation with 100 mg/kg CB can alleviate intestinal oxidative stress, increase the abundance of beneficial bacteria, and decrease the abundance of harmful bacteria in weaned pigs [16].

Here, we hypothesized that high AA level and dietary supplementation with CB could effectively control the diarrhea of weaned pigs and improve the growth performance of weaned pigs. To test the hypothesis, we assigned 532 weaned pigs to 4 treatments (Low AA, Low AA + carvacrol–thymol blend, High AA, High AA + carvacrol–thymol blend) for 42 d after weaning respectively, and collected the plasma and fecal at d 7, 14, 42 to test effects of different AA levels and a carvacrol–thymol blend on growth performance and intestinal health of weaned pigs.

## Methods

### Animals and experimental design

A total of 532 weaned pigs (Duroc × Large White × Landrace) with an initial BW of 7.74 ± 1.23 kg were randomly allocated to 4 groups with four-five pens (7.1 m × 4.7 m) per treatment and 28 weaned pigs per pen, the stocking density is 1.19 m^2^/pig. Porcine circovirus (PCV), pseudorabies virus (PRV), classical swine fever (CSF), porcine reproductive and respiratory syndrome virus (PRRS) vaccine were injected at 3, 18 and 22 d respectively. The experiment was designed with 2 × 2 factors including a low level AA diet (Low AA), a low level AA diet supplemented with carvacrol–thymol blend (50 mg/kg carvacrol and 50 mg/kg thymol of diet) (Low AA+CB), a high level AA diet (High AA), a high level AA diet supplemented with carvacrol–thymol blend (50 mg/kg carvacrol and 50 mg/kg thymol of diet) (High AA+CB) for 42 d. The level of SID Lys of low AA group from d 1 to d 14 is 1.35%, and then change to 1.25% from d 15 to d 42. The level of SID Lys of high AA group from d 1 to d 14 is 1.50%, and then change to 1.40% from d 15 to d 42. The carvacrol–thymol blend was provided by Novus International Inc. (St. Louis, MO, USA) as Next Enhance 150® (1:1, Thymol:Carvacrol). According to the manufacturer, Next Enhance 150 contains 50% encapsulated active components (thymol and carvacrol) but no other nutrients. The composition and nutrient level of the basal diet are shown in Tables 1 and 2. All of the piglets were given ad libitum access to water and feed.

**Table 1:** Ingredients and nutrient composition of diet during 1 ~ 14 d

Composition, %	Low AA		High AA	
	CB(−)	CB(+)	CB(−)	CB(+)
Prepuffed raw materials	53.08	53.03	54.16	54.11
Fermented soybean meal	8.90	8.90	7.40	7.40
Yeast	2.08	2.08	2.08	2.08
Fermented products	6.67	6.67	6.67	6.67
Low protein whey powder	12.50	12.50	12.50	12.50
High protein whey powder	2.78	2.78	2.78	2.78
Glucose	2.78	2.78	2.78	2.78
Sucrose	2.50	2.50	2.50	2.50
Soybean oil	2.60	2.60	2.30	2.30
Phospholipid powder	1.50	1.50	1.50	1.50
Limestone	0.29	0.29	0.29	0.29
Dicalcium phosphate	0.67	0.67	0.69	0.69
Sodium chloride	0.23	0.23	0.23	0.23
Lysine, 98%	0.57	0.57	0.8	0.8
Methionine	0.25	0.25	0.35	0.35
Threonine	0.31	0.31	0.42	0.42
Tryptophan	0.10	0.10	0.14	0.14
Valine	0.18	0.18	0.30	0.30
Isoleucine	0.09	0.09	0.19	0.19
ZnO	0.20	0.20	0.20	0.20
Activate® DA	0.50	0.50	0.50	0.50
Montmorillonite	0.40	0.40	0.40	0.40
Feed antifungal agent	0.05	0.05	0.05	0.05
Choline chloride	0.12	0.12	0.12	0.12
Food attractant	0.05	0.05	0.05	0.05
Premix*	0.60	0.60	0.60	0.60
Carvacrol and thymol		0.05		0.05
Total	100	100	100	100
Nutrient content				
Digestible energy, kcal/kg	3463.00	3463.00	3458.60	3458.60
Crude protein, %	18.00	18.00	17.98	17.98
Digestible lysine, %	1.35	1.35	1.50	1.50
Digestible methionine, %	0.62	0.62	0.71	0.71
Digestible threonine, %	0.88	0.88	0.97	0.97
Digestible tryptophan, %	0.27	0.27	0.30	0.30
Digestible valine, %	0.87	0.87	0.96	0.96
Ca, %	0.50	0.50	0.49	0.49
Available P, %	0.36	0.36	0.36	0.36

*Provided per kg of diet: Vitamin E 200 mg, Vitamin C 350 mg, piglet multidimensional 350 mg, piglet micromine 3000 mg, protein enzyme (Cibenza® DP100) 100 mg, phytase 200 mg, microecological preparations 300 mg, antiseptic 1000 mg, antioxidants 200 mg, flavour 800 mg, sweetener 300 mg, sodium butyrate 1500 mg

**Table 2:** Ingredients and nutrient composition of diet during 15 ~ 42 d

Composition, %	Low AA		High AA	
	CB (−)	CB(+)	CB(−)	CB(+)
Corn	30.40	30.35	31.40	31.35
Prepuffed raw materials	33.04	33.04	32.99	32.99
Pretreatment raw materials	7.00	7.00	7.00	7.00
Soybean meal, 46% CP	5.40	5.40	4.00	4.00
Fermented soybean meal	8.00	8.00	8.00	8.00
Brewer’s yeast hydrolysate	1.39	1.39	1.39	1.39
Low protein whey powder	5.56	5.56	5.56	5.56
High protein whey powder	1.39	1.39	1.39	1.39
Cheese whey	0.69	0.69	0.69	0.69
Soybean oil	1.90	1.90	1.60	1.60
Phospholipid powder	0.50	0.50	0.50	0.50
Limestone	0.80	0.80	0.80	0.80
Dicalcium phosphate	0.82	0.82	0.84	0.84
Sodium chloride	0.39	0.39	0.39	0.39
Lysine, 98%	0.58	0.58	0.82	0.82
Methionine	0.17	0.17	0.27	0.27
Threonine	0.26	0.26	0.38	0.38
Tryptophan	0.08	0.08	0.12	0.12
Valine	0.13	0.13	0.25	0.25
Isoleucine	0.03	0.03	0.14	0.14
Feed antifungal agent	0.05	0.05	0.05	0.05
Montmorillonite	0.40	0.40	0.40	0.40
Choline chloride	0.1	0.1	0.1	0.1
Food attractant	0.05	0.05	0.05	0.05
Activate® DA	0.27	0.27	0.27	0.27
Premix *	0.60	0.60	0.60	0.60
Carvacrol and thymol		0.05		0.05
Total	100	100	100	100
Nutrient content				
Digestible energy, kcal/kg	3427.70	3427.70	3425.10	3425.10
Crude protein, %	17.99	17.99	17.98	17.98
Digestible lysine, %	1.25	1.25	1.40	1.40
Digestible methionine, %	0.49	0.49	0.58	0.58
Digestible threonine, %	0.81	0.81	0.91	0.91
Digestible tryptophan, %	0.25	0.25	0.28	0.28
Digestible valine, %	0.83	0.83	0.92	0.92
Ca, %	0.60	0.60	0.59	0.59
Available P, %	0.34	0.34	0.34	0.34

*Provided per kg of diet: Vitamin E 200 mg, Vitamin C 350 mg, piglet multidimensional 350 mg, piglet micromine 3000 mg, protein enzyme (Cibenza® DP100) 100 mg, phytase 200 mg, microecological preparations 300 mg, antiseptic 1000 mg, antioxidants 200 mg, flavour 800 mg, sweetener 300 mg, sodium butyrate 1500 mg

### Determinations of growth performance and diarrhea

The body weight (BW) of the pigs were recorded at d 1, d 14 and d 42; Feed intake of weaned pigs was recorded every day, and average daily feed intake (ADFI), average daily gain (ADG), and FCR were calculated per pen. The diarrhea rate was recorded during d 1~14 after weaning, and the severity of diarrhea was evaluated by using the fecal consistency score system. In brief, scores were 0, firm, normal; 1, pasty; slight diarrhea; 2, semi-liquid, moderate diarrhea; or 3, liquid and unformed, severe diarrhea [19, 20]. The diarrhea rate was calculated as follows: Diarrhea rate (%) = (number of diarrhea weaned pigs)/(total number of experimental weaned pigs × experimental time (d)) × 100%. Diarrhea index = total fecal scores/total number of experimental weaned pigs.

### Sample collections

At d 7 and 14, 2 pigs per pen were randomly selected for blood samples via the anterior vena cava puncture (tubes containing heparin sodium) and plasma was obtained after centrifugation at 900 × *g* for 10 min. The fresh fecal samples of the same weaned pigs were collected at the same time. Samples were frozen at − 80 °C until analysis.

### Plasma and fecal chemical analysis

0.2 g of feces was weighed and put in the 1.5-mL EP tube, 0.8 mL PBS was added, and it was homogenized on the mixer. After that, it was centrifuged at 400 × *g* at 4 °C for 5 min, and the supernatant was collected.

The activities of total superoxide dismutase (T-SOD), glutathione peroxidase (GSH-px), the plasma concentrations of malondialdehyde (MDA), antioxidative capacity (T-AOC) and plasma urea nitrogen (PUN) were detected using colorimetric methods with a spectrophotometer (Biomate 5, Rochester, NY, USA). The assays were carried out using commercial kits (Nanjing Jiancheng Bioengineering Institute, Nanjing, Jiangsu, China) and their corresponding procedures. The assays were performed in triplicate.

The level of plasma tumor necrosis factor α (TNF-α), IL-1β, IL-6, Interleukin 8 (IL-8) and the level of fecal Lipoprotein 2 were measured to evaluate the systemic and intestinal inflammatory response. The level of plasma endotoxin, D-lactate, diamine oxidase (DAO), and the level of fecal sIgA (P-KMLJ939189), MUC 2 were measured to evaluate the gut barrier function of weaned pigs. Pig TNF-α ELISA kit (2P-KMLJ942147p), IL-1β ELISA kit (2P-KMLJ941952p), IL-6 ELISA kit (P-KMLJ941958p), IL-8 ELISA kit (2P-KMLJ941959p), Lipoprotein 2 (2P-KMLJ942831p), Endotoxin (2P-KMLJ942056p), DAO (2P-KMLJ942522p), D-lactate (2P-KMLJ942838p), sIgA (2P-KMLJ941995p), MUC 2 (2P-KMLJ942137p) were purchased from Nanjing Camilo biological engineering Co., Ltd. (Camilo, Nanjing, China). Briefly, 10 μL of plasma or fecal supernatant was added in the microplate. Add HRP-antibody solution and incubate at 37 °C. Then wash and add substrate solution. Incubate at 37 °C in the dark. Add stop solution and measure absorbance at 450 nm.

### Determinations of fecal SCFAs

Fecal concentrations of short chain fatty acids (SCFAs) were determined as previously described [21], with slight modifications. In brief, approximately 0.1 g of fecal samples (*n* = 8–10/group) was placed into 1.5-mL centrifuge tubes, diluted with 1 mL 0.5% of phosphoric acid solution and homogenized. Then, the samples were centrifuged at 14,400 × *g* for 10 min to obtain the supernatant. The supernatant was extracted with equal volume of ethyl acetate, and precipitated in refrigerator at − 20 °C. Then, the samples were centrifuged at 14,400 × *g* for 10 min to obtain the supernatant. The concentrations of SCFAs in the supernatant were determined using gas chromatography (Thermo, Waltham, USA). All procedures were performed in duplicate.

### DNA extraction and real-time quantitative polymerase chain reaction (PCR)

Total microbial DNA was extracted and purified from fecal samples on d 14 and 42 using a QIAamp DNA stool kit (TIANGEN, Beijing, China) according to the manufacturer’s instructions. The quantity and quality of DNA was assessed using a NanoDropfi ND-1000 Spectrophotometer. Real-time quantitative polymerase chain reaction (PCR) analyses were performed by CFX ConnectTM Real-time PCR Detection System (Bio-Rad, Hercules, USA) in a final reaction volume of 10 μL containing 4.4 μL of template DNA (50 ng/μL), 5 μL iTaq™ Universal SYBR Green Supermix (Bio-Rad, Hercules, USA) and 0.3 μL of each of forward and reverse primers. Thermal cycling conditions involved an initial denaturation step at 95 °C for 10 min followed by 40 cycles of denaturation at 95 °C for 15 s and 65 °C for 1 min [22]. Dissociation analyses of the PCR product were performed to confirm the specificity of the resulting PCR products. The primers used for the real-time PCR detection of selected genes are listed in Table 3.

**Table 3:** Primers used for absolute quantitative real-time polymerase chain reaction (PCR)

Target group	Sequence of primers, 5′ to 3′	Size, bp
Total bacteria	Forward: GTGSTGCAYGGYYGTCGTCA	146
	Reverse: ACGTCRTCCMCNCCTTCCTC	
*Lactobacillus* spp.	Forward: AGCAGTAGGGAATCTTCCA	341
	Reverse: CACCGCTACACATGGAG	
*Enterococcus*	Forward: CCCTTATTGTTAGTTGCCATCATT	144
	Reverse: ACTCGTTGTACTTCCCATTGT	
*Escherichia coli*	Forward: CATGCCGCGTGTATGAAGAA	96
	Reverse: CGGGTAACGTCAATGAGCAAA	
*Bifidobacterium* genus	Forward: TCGCGTCTGGTGTGAAAG	243
	Reverse: CCACA TCCAGCATCCAC	

### Statistical analysis

All the data were analyzed using the general linear model (GLM) procedure of the Statistical Analysis. System (SAS 9.2 SAS Institute Inc., Cary, NC, USA). Data of the animal trial were statistically analyzed using the two-factor ANOVA to determine the main effects of dietary AA level, CB supplementation, and their interactions. If there was a main effect or the interaction was significant, the Bonferroni *t*-test was performed for post hoc comparison of means. The χ^2^ test was used to test for diarrhea rate. Data were expressed as means ± standard error of the mean (SEM). Level of significance was set at *P* < 0.05, whereas 0.05 < *P* < 0.1 was considered a trend towards significance.

## Results

### Growth performance and diarrhea

The growth performance and diarrhea rate of weaned pigs are shown in Table 4. There was not a significant interaction effect between AA level and CB supplementation on the growth performance (*P* > 0.05). During d 1~14 after weaning, high AA level significantly decreased ADFI and FCR of weaned pigs (*P* < 0.05). Dietary supplementation with CB significantly increased the final weight, ADG, ADFI and average daily lysine intake (ADLysI) (*P* < 0.05). There was a significant interaction effect between AA level and CB supplementation on the diarrhea rate (*P* < 0.05). During 15~42 d and 1~42 d after weaning, there was no significant difference in growth performance among different treatment groups.

**Table 4:** Growth performance of piglets

Items	Low AA		High AA		SEM	*P*-value		
	CB (−)	CB(+)	CB(−)	CB(+)		AA	CB	AA×CB
Number of pens	5	5	4	5				
1 d BW, kg	7.74	7.75	7.74	7.77	0.01	0.60	0.40	0.91
14 d BW, kg	10.85	11.29	11.07	11.56	0.09	0.14	< 0.01	0.87
42 d BW, kg	29.38	29.34	28.87	29.14	0.32	0.56	0.85	0.80
1 **~** 14 d								
ADG, g/d	226.49	255.57	236.61	268.09	6.33	0.32	0.01	0.91
ADFI, g/d	340.91	372.02	311.59	343.63	8.41	0.04	0.03	0.97
ADLysI, g/d	4.60	5.02	4.67	5.15	0.10	0.58	0.02	0.87
FCR	1.51	1.46	1.32	1.29	0.03	< 0.01	0.31	0.91
Diarrhea rate, %	7.31 ^a^	5.78 ^ab^	5.51 ^ab^	5.24 ^b^	0.47	0.03	0.07	0.03
Diarrhea index	0.18 ± 0.04	0.15	0.13	0.12	0.01	0.09	0.64	0.46
15 **~** 42 d								
ADG, g/d	657.21	642.87	632.62	628.98	8.28	0.28	0.61	0.76
ADFI, g/d	899.44	905.25	899.84	888.96	16.46	0.82	0.94	0.81
ADLysI, g/d	11.24	11.32	12.6	12.45	0.25	0.01	0.93	0.80
FCR	1.37	1.41	1.42	1.41	0.01	0.98	0.72	0.39
1 **~** 42 d								
ADG, g/d	515.35	514.56	501.96	508.79	6.47	0.50	0.83	0.79
ADFI, g/d	713.27	727.51	703.76	707.19	12.92	0.57	0.74	0.84
ADLysI, g/d	7.92	8.17	8.64	8.80	0.15	0.03	0.48	0.89
FCR	1.38	1.41	1.40	1.39	0.01	0.98	0.72	0.39

All results are presented as mean ± SEM. *AA* Amino acid effect, *CB* a carvacrol–thymol blend effect, *AA×CB* interaction effect of amino acid and a carvacrol–thymol blend, *ADLysI* average daily lysine intake. *P* < 0.05 significant difference, *P* < 0.01 extremely significant difference

### The content of plasma urea nitrogen

**Fig. 1:**
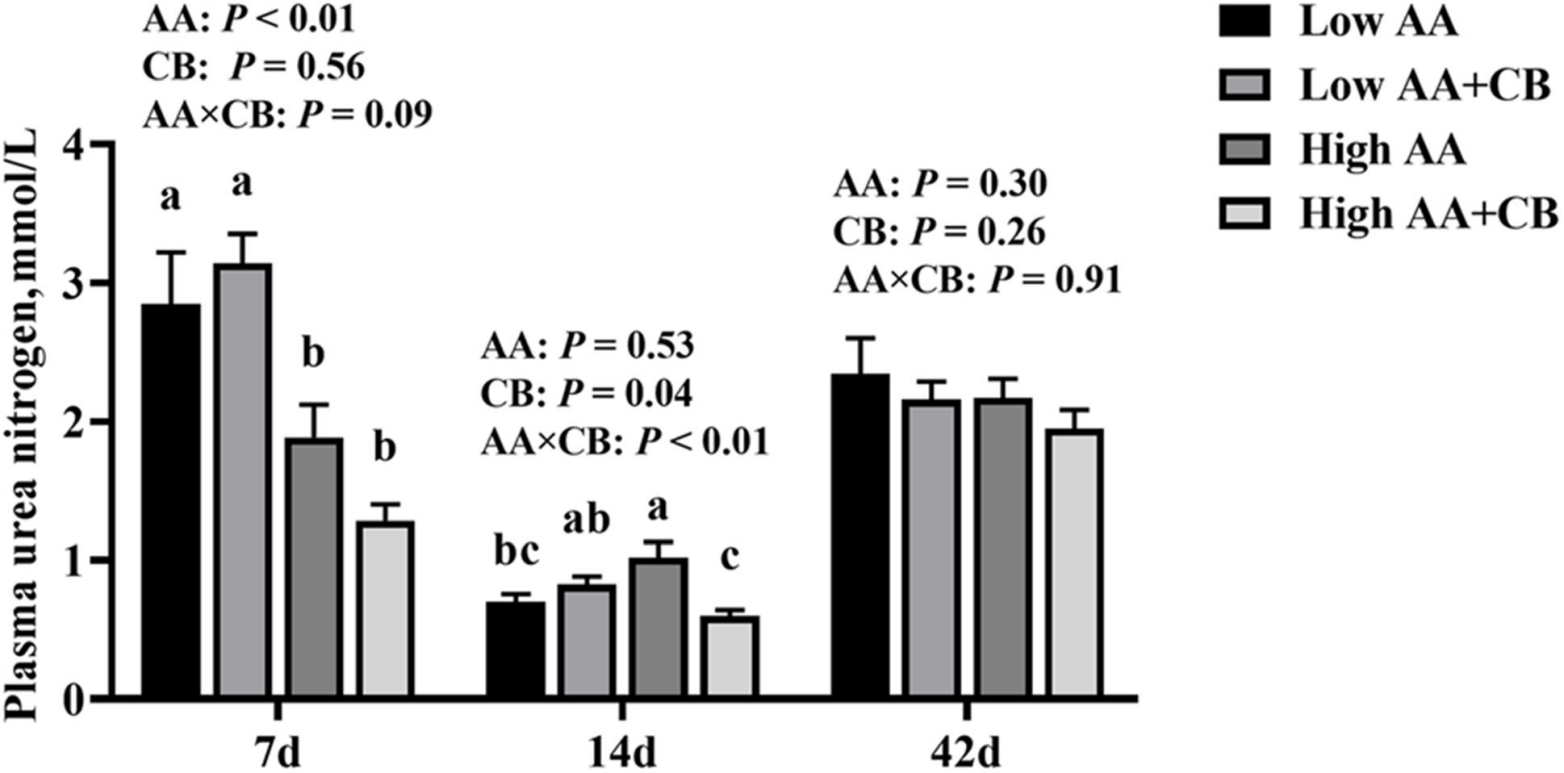
Plasma urea nitrogen content in piglets. All results are presented as mean ± SEM (*n* = 8–10/group). AA: Amino acid effect; CB: a carvacrol–thymol blend effect, AA×CB: interaction effect of amino acid and a carvacrol–thymol blend. *P* < 0.05 significant difference, *P* < 0.01 extremely significant difference

As shown in Fig. 1, at d 14 after weaning, there was an interaction between AA level and CB supplementation on the content of PUN in weaned pigs (*P* < 0.05), which showed that high level AA dietary supplemented with CB significantly reduced the content of PUN (*P* < 0.05), but not compared to low AA (*P* > 0.05). At d 7 after weaning, high AA level significantly (*P* < 0.05) reduced the content of PUN.

### Antioxidant activity and lipid peroxidation in plasma

As shown in Table 5. There was no interaction between AA level and a carvacrol–thymol blend on antioxidant enzyme activity and MDA content in plasma of weaned pigs. Dietary supplementation with CB significantly (*P* < 0.05) increased the plasma T-SOD activity at d 7 after weaning and GSH-px activity at d 14 after weaning, and significantly decreased the plasma MDA content at d 7 and d 14 after weaning (*P* < 0.05). Different AA levels had no significant (*P* > 0.05) effects on antioxidant enzyme activity and MDA content in plasma of weaned pigs.

**Table 5:** Antioxidant enzyme activity and MDA content in plasma of piglets

Times	Items	Low AA		High AA		SEM	*P*-value		
		CB (−)	CB (+)	CB (−)	CB (+)		AA	CB	AA×CB
	Number of pens	10	10	8	10				
7 d	T-SOD, U/mL	25.94	37.39	33.18	40.07	1.83	0.15	0.01	0.50
	GSH-px, μmol/L	1023.54	992.86	998.93	935.54	20.77	0.34	0.27	0.70
	MDA, nmol/mL	6.96	5.81	7.33	6.52	0.24	0.26	0.04	0.72
	T-AOC, U/mL	0.76	0.75	0.75	0.79	0.01	0.38	0.59	0.12
14 d	T-SOD, U/mL	42.37	38.33	35.58	37.69	1.28	0.16	0.71	0.24
	GSH-px, μmol/L	576.94	644.94	610.51	694.28	16.94	0.20	0.02	0.81
	MDA, nmol/mL	7.35	5.52	7.52	6.77	0.27	0.17	0.02	0.30
	T-AOC, U/mL	0.71	0.71	0.71	0.7	0.01	1.00	0.33	0.83

All results are presented as mean ± SEM. *AA* Amino acid effect, *CB* a carvacrol–thymol blend effect, *AA×CB* interaction effect of amino acid and a carvacrol–thymol blend. *P* < 0.05 significant difference, *P* < 0.01 extremely significant difference

### Level of inflammatory factors in plasma and feces

**Fig. 2:**
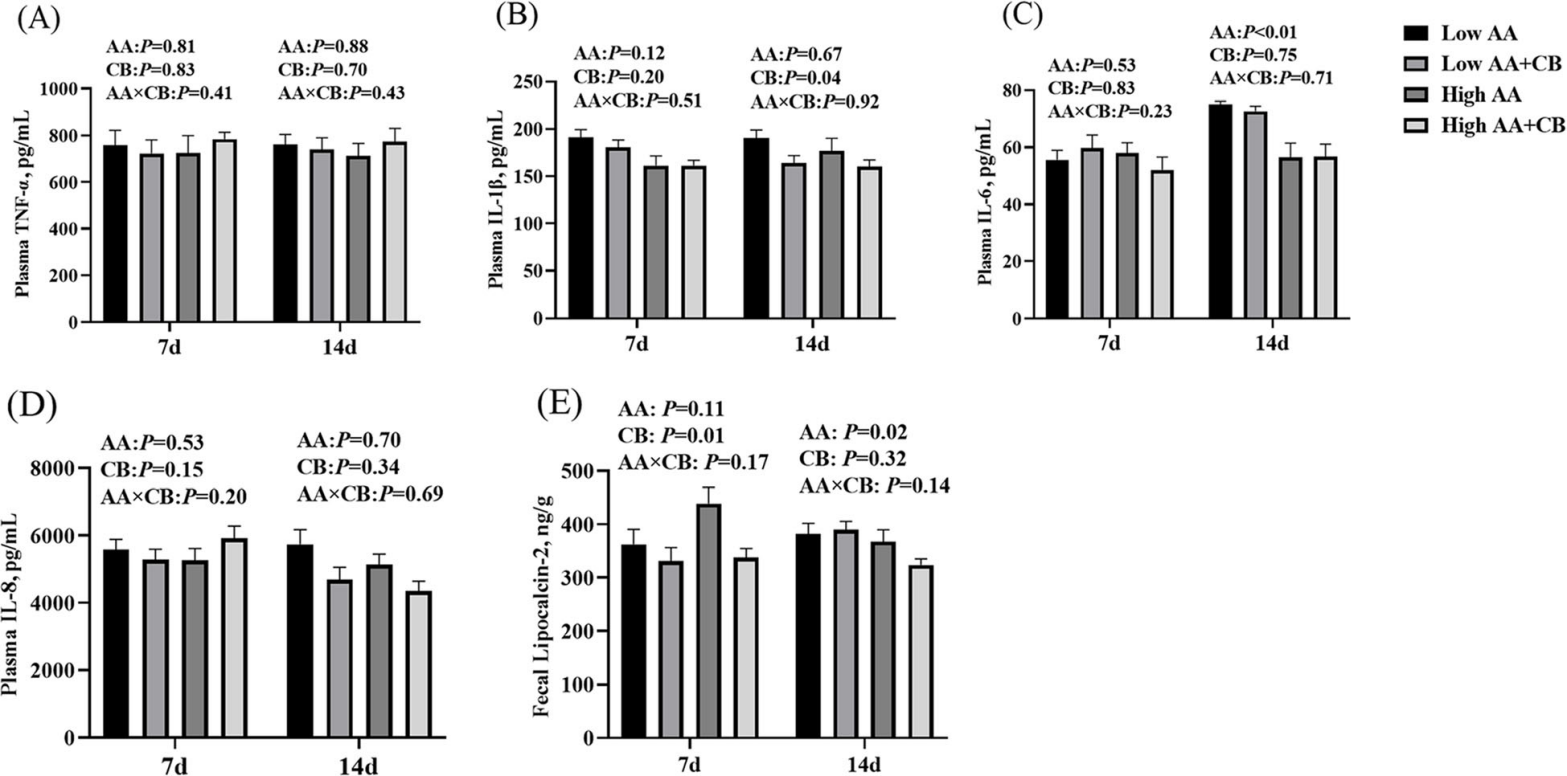
Effects of different treatments on inflammatory factors in piglets. **A** The content of TNF-α in plasma; **B** the content of IL-1β in plasma; **C** the content of IL-6 in plasma; **D** the content of IL-8 in plasma; **E** the content of Lipocalcin-2 in fecal. All results are presented as mean ± SEM (*n* = 8–10/group). AA: Amino acid effect; CB: a carvacrol–thymol blend, AA×CB: interaction effect of amino acid and a carvacrol–thymol blend. *P* < 0.05 significant difference, *P* < 0.01 extremely significant difference

As shown in Fig. 2. There was no interaction between AA level and a carvacrol–thymol blend on the content of inflammatory factors in weaned pigs. High AA level significantly reduced the content of plasma IL-6 and fecal lipoprotein-2 at d 14 after weaning (Fig. 2C & E) (*P* < 0.05); Dietary supplementation with CB significantly reduced the content of fecal lipoprotein 2 at d 7 after weaning and plasma IL-1β at d 14 after weaning (Fig. 2B & E) (*P* < 0.05).

### Selected fecal bacterial populations

**Fig. 3:**
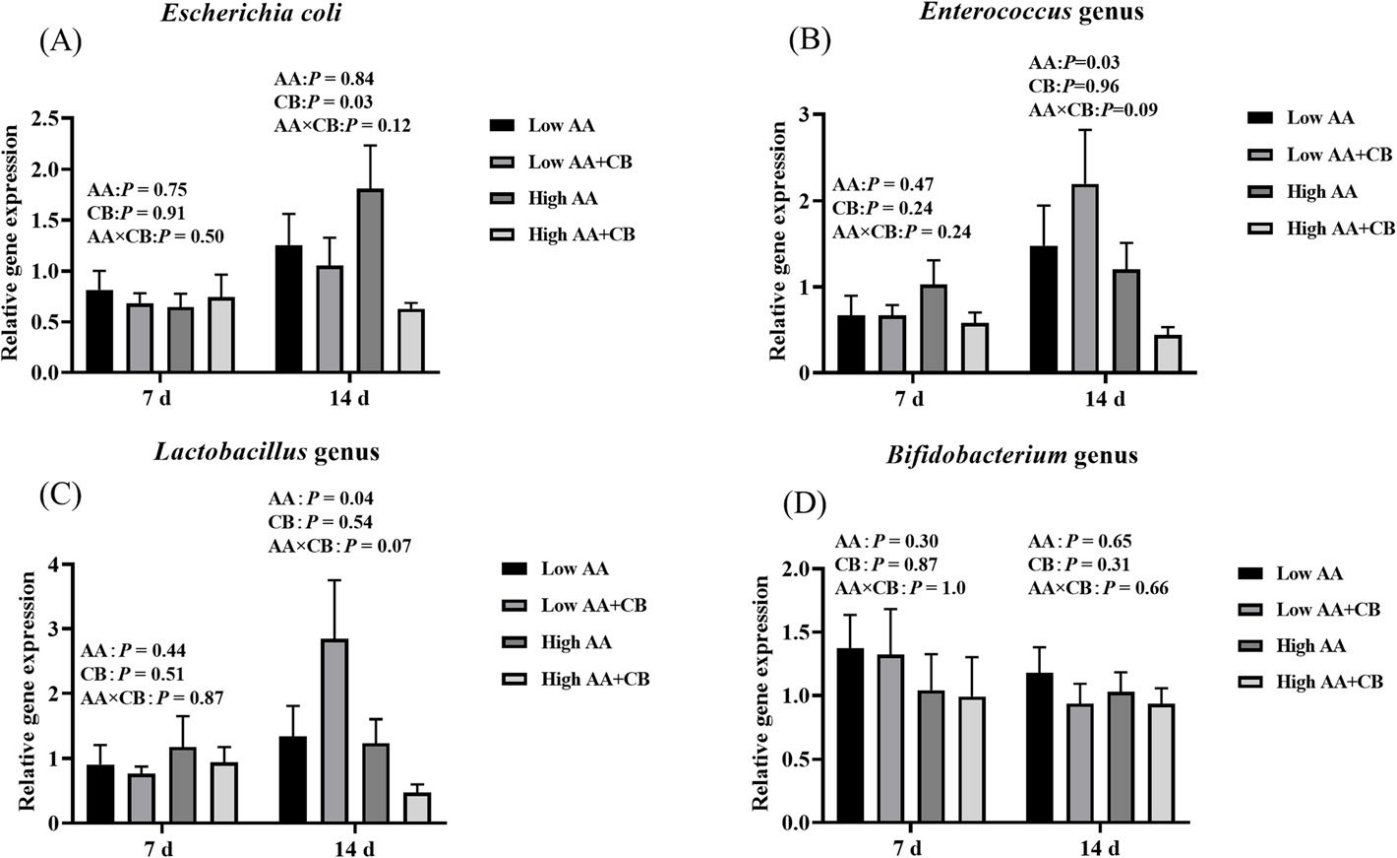
Effects of different treatments on specific microorganisms in piglet feces. Relative abundance of **A** *E. coli*, **B** *Enterococcus*, **C** *Lactobacillus* and **D** *Bifidobacterium* in piglet feces. All results are presented as mean ± SEM (*n* = 8–10/group). AA: Amino acid effect; CB: a carvacrol–thymol blend effect, AA×CB: interaction effect of amino acid and a carvacrol–thymol blend. *P* < 0.1 indicates the tendency of significant difference, *P* < 0.05 significant difference

As shown in Fig. 3. At d 14 after weaning, increasing dietary AA level significantly increased the relative abundance of fecal *Enterococcus* and *Lactobacillus* (*P* < 0.05) (Fig. 3B & C); Dietary supplementation with CB significantly reduced the relative abundance of fecal *Escherichia coli* (*P* < 0.05) (Fig. 3A). Whereas, there are not significant effects on selected fecal bacterial populations with high AA level and dietary supplementation with CB at d 7 after weaning (*P* > 0.05) (Fig. 3).

### Fecal SCFAs

**Fig. 4:**
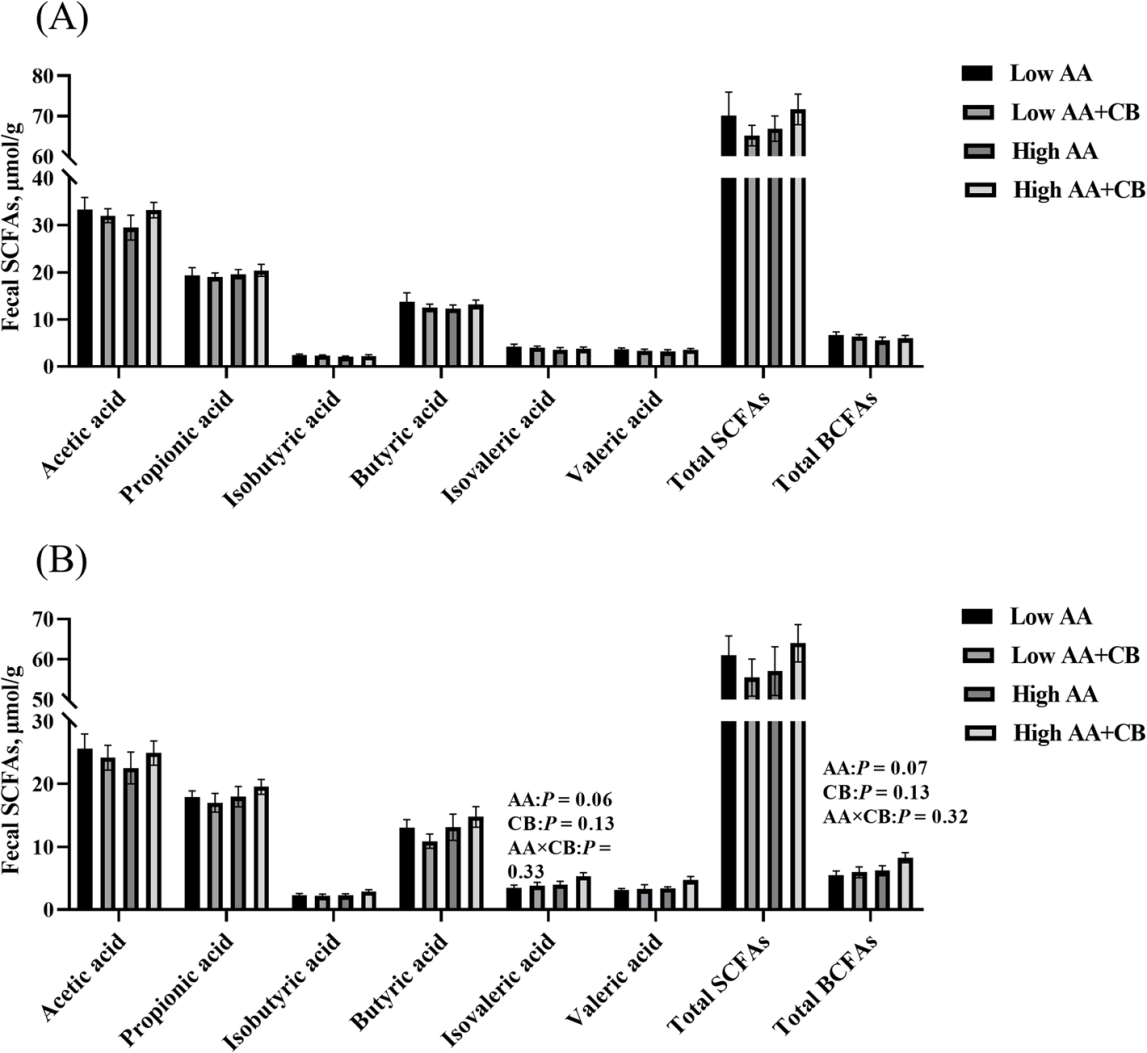
Concentrations of SCFAs in piglet feces. **A** The content of SCFAs in feces of piglets on d 7 after weaning; **B** the content of SCFAs in feces of piglets on d 14 after weaning. All results are presented as mean ± SEM (*n* = 8–10/group). AA: Amino acid effect; CB: a carvacrol–thymol blend effect, AA×CB: interaction effect of amino acid and a carvacrol–thymol blend. *P* < 0.1 indicates the tendency of significant difference

As shown in Fig. 4. There was no interaction between AA level and a carvacrol–thymol blend on the content of SCFAs in piglet feces. High AA level tended to increase the content of isovaleric acid and total branched chain fatty acids (isobutyric acid and isovaleric acid) at d 14 after weaning (Fig. 4B) (*P* < 0.1). Dietary supplementation with CB had no significant (*P* > 0.05) effects on the content of SCFAs in piglet feces (Fig. 4).

### Biomarkers of intestinal barrier function

**Fig. 5:**
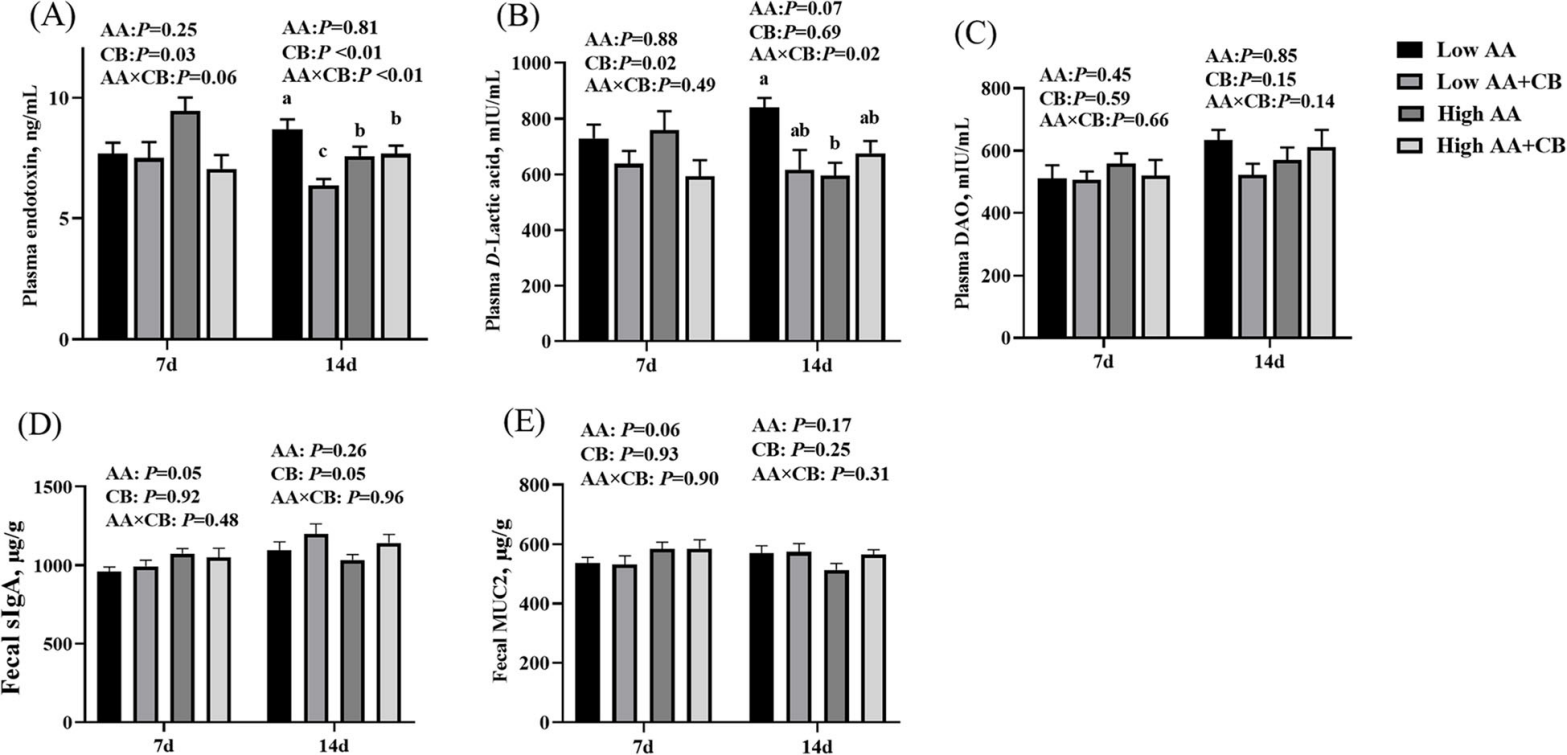
Effects of different treatments on intestinal barrier function in piglets. **A** The content of endotoxin in plasma; **B** the content of *D*-lactic acid in plasma; **C** the content of DAO in plasma; **D** the content of secretory immunoglobulin A content in fecal; **E** the content of MUC 2 in fecal. All results are presented as mean ± SEM (*n* = 8–10/group). AA: Amino acid effect; CB: a carvacrol–thymol blend effect, AA×CB: interaction effect of amino acid and a carvacrol–thymol blend. *P* < 0.1 indicates the tendency of significant difference, *P* < 0.05 significant difference, *P* < 0.01 extremely significant difference

The biomarkers of intestinal barrier function were shown in Fig. 5. At d 7 after weaning, there was a trend of interaction between AA level and a carvacrol–thymol blend on endotoxin content in plasma (Fig. 5A) (*P* < 0.1). Dietary supplementation with CB significantly decreased plasma endotoxin at low AA level (*P* < 0.05). High AA level had a tendency to increase fecal sIgA and fecal MUC 2 contents at d 7 after weaning (Fig. 5D & E) (*P* < 0.1). Dietary supplementation with CB significantly reduced plasma endotoxin and *D*-lactic acid content at d 7, 14 and d 7 after weaning respectively (*P* < 0.05) (Fig. 5A & B). At d 14 after weaning, there was an interaction between AA level and a carvacrol–thymol blend on plasma endotoxin and *D*-lactic acid content (Fig. 5A & B) (*P* < 0.05). Low level of AA diet supplemented with CB significantly reduced the content of endotoxin in plasma at d 14 after weaning (*P* < 0.01) (Fig. 5A). Dietary supplementation with CB had a tendency to increase the content of sIgA in feces at d 14 after weaning (*P* = 0.05) (Fig. 5D).

## Discussion

Essential AA are precursors of many bioactive substances and play an important role in promoting the growth of animals [23, 24]. High AA level significantly reduced ADFI and FCR of weaned pigs, which may be related to the transport of AA. Studies have shown that reducing dietary lysine level inhibited the expression of lysine transporter and the transport of lysine in the intestine, which promoted the feed uptake of piglets [25]. The decrease of FCR during the first two weeks postweaning may be related to the decrease of PUN content. PUN is an important index to evaluate the nitrogen utilization rate, the lower level of PUN, the higher the nitrogen utilization rate of animal body in protein synthesis [26, 27]. Therefore, increasing the level of AA may improve the FCR of weaned pigs by increasing the utilization rate of AA.

Our results showed that dietary supplementation CB significantly increased BW, ADG, ADLysI and ADFI of weaned pigs, but had no significant effect on FCR of weaned pigs. We hypothesis dietary supplementation CB decreased the content of PUN to elevated the utilize of AA in piglet thus to promote the growth performance. Other studies also demonstrated that the content of PUN in weaned pigs was significantly reduced by feeding carvacrol and thymol diets [18]. In addition, this study found that dietary supplementation CB improved the growth performance of weaned pigs primarily in the early stage of the experiment, probably because the effects of weaning on the intestinal structure of weaned pigs was mainly in the early stage of weaning [28].

Early weaning pigs are affected by diet changes, environmental, physiological and psychological factors, are prone to slow growth, diarrhea, intestinal barrier function damage and other weaning stress problems, which may have negative effects on the health and growth of weaned pigs [29–31]. The increasing content of plasma endotoxin and *D*-lactic acid are a marker of increased intestinal permeability. Endotoxin as a component of the exterior cell wall of Gram-negative bacteria, the abundance of endotoxin in plasma presents enhanced intestinal permeability and poor intestinal barrier function [32, 33]. *D*-lactic acid is a metabolic product of bacterial fermentation, which can be produced by a variety of intestinal bacteria. When intestinal mucosal permeability increases, a large amount of *D*-lactic acid produced by intestinal bacteria enters the blood through the damaged mucosa, increasing the serum *D*-lactic acid level [34, 35]. In the present study, supplementation with CB decreased plasma concentrations of endotoxins and *D*-lactate at d 7, 14 and d 7 after weaning respectively. At d 14 after weaning, there was an interaction between AA level and a carvacrol–thymol blend on plasma endotoxin and *D*-lactic acid content. These results indicated that the effects of CB on intestinal permeability of weaned pigs are not consistent at different AA levels, and there may be obvious differences of mechanisms.

Adequate AA intake is particularly important for intestinal physiology of weaned pigs [36]. We found that high AA level significantly reduced fecal lipoprotein-2 content, and had a tendency to increase fecal MUC 2 and sIgA. Fecal Lipocalin 2 is a biomarker for intestinal inflammation [37]. Mucin secreted by goblet cells, especially MUC 2 plays an important role in maintaining intestinal mucosal barrier function [38]. In the small intestine, sIgA can directly reflect the immune barrier function of intestinal mucosa [39]. These results indicated that dietary AA levels might alleviate the intestinal inflammatory reaction, improved the intestinal immune and chemical barrier function of weaned pigs.

Intestinal oxidative stress and inflammatory response during weaning are one of the reasons for the impairment of intestinal barrier function [40, 41]. The results showed that dietary supplementation with CB significantly alleviated the oxidative stress induced by weaning. Carvacrol and thymol have strong antioxidant activity and play an important role in scavenging free radicals and peroxynitrite and inhibiting lipid peroxidation [42, 43]. The phenolic hydroxyl groups contained in carvacrol and thymol can act as hydrogen donors to bind with peroxy radical and block the oxidative chain reaction, thus preventing and delaying lipid oxidation [44]. Carvacrol and thymol are the main role in the antioxidant function in oragno essienal oil [45], Our previous studies also found that dietary supplementation with CB or oragno essienal oil had antioxidant function in piglets [16], growing finishing pigs [46], sows [47] and boars [48]. In addition, we also found that the relative abundance of fecal *Escherichia coli* was significantly reduced by dietary supplementation with CB. Dietary supplementation with CB can reduce the relative abundance of free radical producing bacteria such as *Escherichia coli* in piglets’ intestines [16]. The deamination of valine and leucine by bacteria produces isobutyric acid and isoprene, which are the markers of protein fermentation [49]. Although high AA level had the tendency to increase the fecal isoprene and total branched chain fatty acids, but it did not increase diarrhea or damage the intestinal health of weaned pigs. Therefore, dietary supplementation with CB improved intestinal permeability may be related to alleviating oxidative stress in weaned pigs.

## Conclusion

The improvement of dietary AA level mainly affected the utilization of AA in early stage after weaning, and improved the intestinal barrier function of weaned pigs, which could alleviate diarrhea and promote the growth of weaned pigs. Dietary supplementation with CB could reduce the relative abundance of harmful bacteria and improve the integrity of intestinal structure of weaned pigs by reducing oxidative stress and inflammatory response of the body, and then promoting the growth of weaned pigs. The weaned pigs will reach the best growth rate in 2 weeks after weaning by increasing the level of dietary Lys at 1.5% and 1.4% together with supplementation with CB.

## Data Availability

The datasets used and/or analysed during the current study are available from the corresponding author on reasonable request.

## Abbreviations

ADG: Average daily gain
ADFI: Average daily feed intake
BCFAs: Branched chain fatty acid
CB: Carvacrol–thymol blend
DAO: Diamineoxidase
FCR: Feed conversion ratio
GSH-PX: Glutathione peroxidase
IL-1β: Interleukin-1β
IL-6: Interleukin-6
IL-8: Interleukin-8
IGF: Insulin-like growth factor
MCFAs: Medium chain fatty acids
MDA: Malonaldehyde
MUC 2: Mucin 2
OAs: Organic acids
PUN: Plasma urea nitrogen
SCFAs: Short chain fatty acids
sIgA: Secreted immunoglobin A
